# Numerical Algorithm Based on Haar-Sinc Collocation Method for Solving the Hyperbolic PDEs

**DOI:** 10.1155/2014/340752

**Published:** 2014-11-16

**Authors:** A. Pirkhedri, H. H. S. Javadi, H. R. Navidi

**Affiliations:** ^1^Department of Computer Engineering, Islamic Azad University, Science and Research Branch, Tehran, Iran; ^2^Department of Applied Mathematics, Faculty of Mathematics and Computer Science, Shahed University, Tehran, Iran

## Abstract

The present study investigates the Haar-Sinc collocation method for the solution of the hyperbolic partial telegraph equations. The advantages of this technique are that not only is the convergence rate of Sinc approximation exponential but the computational speed also is high due to the use of the Haar operational matrices. This technique is used to convert the problem to the solution of linear algebraic equations via expanding the required approximation based on the elements of Sinc functions in space and Haar functions in time with unknown coefficients. To analyze the efficiency, precision, and performance of the proposed method, we presented four examples through which our claim was confirmed.

## 1. Introduction

In recent years, the field of hyperbolic partial differential equations has attracted the attention of scientists in several areas and has also been used to solve many actual problems modeled in mathematical physics, such as the vibrations of structures (e.g., buildings, beams, and machines), fluid mechanics, and atomic physics [1].

The telegraph equation has typically been used for transmission and propagation of electrical signals [2], wave propagation model [3], random walk theory [4], and so forth. Let us consider an infinitesimal piece of a telegraph wire which consists of resistor of resistance *R*, a coil of inductance *L*, a resistor of conductance *G*, and a capacitor of capacitance *C*. The telegraph equation is concerned with the changes in voltage between the ends of the piece of the wire which can be formulated as follows [5]:

(1) ∂2y∂t2(x,t)+GC+RL∂y∂t(x,t)+GCRLy(x,t)  =1LC∂2y∂x2(x,t)+f(x,t), (x,t)∈[0,1]×[0,1],

with initial and boundary conditions:

(2) $$\begin{array}{c} y\left(x,0\right)=g_{0}\left(x\right),\;\;\frac{\partial y}{\partial t}\left(x,0\right)=g_{1}\left(x\right), \end{array}$$

(3) $$\begin{array}{c} y\left(0,t\right)=f_{0}\left(t\right),\;\;y\left(1,t\right)=f_{1}\left(t\right), \end{array}$$

where *y*(*x*, *t*) denotes the voltage at position *x* and time *t* and *f*(*x*, *t*) is the external source term. Suppose *ρ* = *G*/*C*, *σ* = *R*/*L* and *η* ^2^ = 1/*LC*; thus, we have

(4) ∂2y∂t2(x,t)+ρ+σ∂y∂t(x,t)+ρσy(x,t)  =η2∂2y∂x2(x,t)+f(x,t).

Because of the lack of appropriate mathematical methods, most of the analytical solutions for linear and nonlinear partial differential equations are challenging to acquire. Therefore, approximation and numerical techniques such as Adomian decomposition method, local radial basis collocation method, variational iteration, homotopy perturbation, and Laplace transform method have been applied [6–14]. The numerical methods for solving the second-order telegraph equation are well developed. Lakestani and Saray [1] solved this problem through expanding the obtained approximate solution as the elements of interpolating scaling functions. Furthermore, the numerical approximation based on differential transform method (DTM) was considered to solve telegraph equation [5]. Using DTM, it is possible to find the exact solution or a closed approximate solution for an equation. The fully discrete local discontinuous Galerkin finite element method based on a finite difference scheme in time was introduced by [15]. Chen et al. [16] used the method of separation of variables for the analytical solution of the nonhomogeneous telegraph equation under three types of nonhomogeneous boundary conditions.

We are inspired to have an algorithm which not only is appropriate for long-time calculations but also reflects the global behavior of exact solutions. The novelty of the present paper is that we investigate the behaviour of the combination of two different groups of orthogonal functions from two different intervals. The combination of piecewise orthogonal Haar functions defined on [0,1) interval, with continuous orthogonal Sinc functions defined on (−*∞*, +*∞*) interval. It is essential to be said that [17–24] have previously used the Haar and Sinc functions separately for solving optimal control problems and some nonlinear ordinary differential equations. For example, Karimi and Lohmann [17] applied the Haar functions for modeling and robust control of bounce and pitch vibration for the enginebody vibration structure. Also, the Haar wavelet method has been investigated for optimal control of time-varying state-delayed [18], linear singularly perturbed systems [19], and second-order linear systems [20]. In addition, based on the properties of orthogonal Sinc functions, it is apparent that the convergence rate of approximation is exponential [21]. By using this property, the authors of [22–24] studied the Sinc collocation method for solving nonlinear singular equations like Thomas-Fermi, Lane-Emden, and Blasius equations.

Another advantage of proposed method is that it transforms the problem into a system of algebraic equations, so that the computation becomes simple and computer oriented. In the new proposed algorithm, we extend the solution of the problem to the sum of basis functions and take good advantage of the orthogonality of Haar and Sinc functions to build a set of equations for the coefficients of the solution. Although the test model is a very simple one, the proposed method is also applicable to many other problems such as fractional and two-dimensional nonlinear PDEs.

The organization of the rest of the paper is as follows. In Section 2, we present a brief introduction to the essential definitions of the Haar and Sinc functions from which are derived some tools for developing our method. In Section 3, the convergence rate analysis of the Haar and Sinc functions is given. In Section 4, we apply the method of Haar-Sinc collocation for solving the model equation. In Section 5, the proposed method is used in some types of telegraph equations and it is compared with the current analytic solutions revealed in different published works within the literature. The conclusion is presented in the final section.

## 2. Basic Definitions

### 2.1. Haar Functions

The orthogonal set of Haar functions is a number of square waves with magnitude of ±1 in some intervals and zeros elsewhere [25]. The Haar functions are defined on the interval *C* = [0, *b*) by

(5) $$\begin{array}{c} h_{u}\left(t\right)=\left\{\begin{array}{cc} 1, & l_{1}\leq t<l_{1/2},\\ -1, & l_{1/2}\leq t<l_{0},\\ 0, & \text{otherwise}, \end{array}\right. \end{array}$$

where,

(6) $$\begin{array}{c} l_{q}=\frac{n-q}{2^{m}}b,\;q=0,\frac{1}{2},1. \end{array}$$

The value of *u* is defined by two parameters *m* and *n* as

(7) $$\begin{array}{c} u=2^{m}+n-1,\;m=0,1,2,\ldots ,M,\;n=1,2,3,\ldots ,2^{m}. \end{array}$$

The integer *m* indicates the level of the wavelet and the maximal level of resolution is the integer *M*. Also, *h*_0_(*t*) is defined for *m* = *n* = 0 and is given by

(8) $$\begin{array}{c} h_{0}\left(t\right)=1,\;0\leq t<b. \end{array}$$

We can expand any function *y*(*t*) ∈ *L* ^2^(0,1) in first *k* terms of Haar functions as

(9) $$\begin{array}{c} y\left(t\right)≃\overset{k-1}{\underset{u=0}{\sum }}x_{u}h_{u}\left(t\right)=X^{T}\phi_{k}\left(t\right), \end{array}$$

where

(10) $$\begin{array}{c} k=2^{s+1},\;s=0,1,2,\ldots . \end{array}$$

The Haar functions coefficient vector *X* and Haar functions vector *ϕ*_*k*_(*t*) are defined as

(11) $$\begin{array}{c} X={\left[x_{0},x_{1},\ldots ,x_{k-1}\right]}^{T}, \end{array}$$

(12) $$\begin{array}{c} \phi_{k}\left(t\right)={\left[h_{0}\left(t\right),h_{1}\left(t\right),\ldots ,h_{k-1}(t)\right]}^{T}. \end{array}$$

The matrix Φ_*k*×*k*_ can be expressed as

(13) $$\begin{array}{c} \Phi_{k\times k}=\left[\begin{array}{c} \phi_{k}\left(\frac{1}{2k}\right),\phi_{k}\left(\frac{3}{2k}\right),\ldots ,\phi_{k}\left(\frac{2k-1}{2k}\right) \end{array}\right]. \end{array}$$

Furthermore, the integration of the *ϕ*_*k*_(*t*) defined in (12) is given by

(14) $$\begin{array}{c} \overset{t}{\underset{0}{\int }}\phi_{k}\left(s\right)ds≃J\phi_{k}\left(t\right), \end{array}$$

where *J* = *J*_*k*×*k*_ is the *k* × *k* operational matrix for integration and is given in [26] as

(15) $$\begin{array}{c} J_{k\times k}=\frac{b}{2k}\left[\begin{array}{cc} 2kJ_{\left(k/2)\times (k/2\right)} & -\Phi_{\left(k/2)\times (k/2\right)}\\ \Phi_{\left(k/2)\times (k/2\right)}^{-1} & 0 \end{array}\right], \end{array}$$

where Φ_1_ = [1], *J*_1_ = [1/2].

### 2.2. Sinc Functions

The Sinc function is defined on the whole real line *I* = (−*∞*, *∞*) by

(16) $$\begin{array}{c} \text{Sinc}\left(x\right)=\left\{\begin{array}{cc} \frac{\sin\left(\pi x\right)}{\pi x}, & x\neq 0,\\ 1, & x=0. \end{array}\right. \end{array}$$

For each integer *m* and the mesh size *h*, the Sinc basis functions are defined on *R* by [22]

(17) Smh,x≡Sincx−mhh=sin⁡π/hx−mhπ/hx−mh,x≠mh,1,x=mh.

The Sinc functions form an interpolatory set of functions; that is,

(18) $$\begin{array}{c} S_{m}\left(h,jh\right)=\delta_{mj}=\left\{\begin{array}{cc} 1, & j=m,\\ 0, & j\neq m. \end{array}\right. \end{array}$$

If a function *f*(*x*) is defined on the real axis, then for *h* > 0 the series,

(19) $$\begin{array}{c} C\left(f,h\right)\left(x\right)=\overset{\infty }{\underset{m=-\infty }{\sum }}f\left(mh\right)\text{Sinc}\left(\frac{x-mh}{h}\right), \end{array}$$

is called the Whittaker cardinal expansion of *f* whenever this series converges. The properties of the Whittaker cardinal expansion have been extensively studied in [27]. These properties are derived in the infinite strip *D*_*S*_ of the complex *ω*-plane, where for *d* > 0,

(20) $$\begin{array}{c} D_{S}=\left\{\omega =t+is:\left|s\right|<d\leq \frac{\pi }{2}\right\}. \end{array}$$

Approximations can be constructed for infinite, semiinfinite, and finite intervals. To construct approximations on the interval (0,1) which is used in this paper, the eye-shaped domain in the* z*-plane,

(21) $$\begin{array}{c} D_{E}=\left\{z=x+iy:\left|\arg\left(\frac{z}{1-z}\right)\right|<d\leq \frac{\pi }{2}\right\}, \end{array}$$

is mapped conformally onto the infinite strip *D*_*S*_ via

(22) $$\begin{array}{c} \omega =\psi \left(z\right)=\ln\left(\frac{z}{1-z}\right). \end{array}$$

The basis functions on (0,1) are taken to be the composite translated Sinc functions:

(23) $$\begin{array}{c} S_{m}\left(x\right)\equiv S\left(m,h\right)\circ \psi \left(x\right)=\text{Sinc}\left(\frac{\psi \left(x\right)-mh}{h}\right), \end{array}$$

where *S*(*m*, *h*)∘*ψ*(*x*) is defined by *S*(*m*, *h*)(*ψ*(*x*)). The inverse map of *ω* = *ψ*(*z*) is

(24) $$\begin{array}{c} z=\psi^{-1}\left(\omega \right)=\frac{e^{\omega }}{1+e^{\omega }}. \end{array}$$

Thus we may define the inverse images of the real line and of the evenly spaced nodes {*mh*}_*m*=−*∞*_^*m*=+*∞*^ as

(25) $$\begin{array}{c} \Gamma =\left\{\psi^{-1}\left(u\right)\in D_{E}:-\infty <u<+\infty \right\}=\left(0,1\right), \end{array}$$

(26) $$\begin{array}{c} x_{m}=\psi^{-1}\left(mh\right)=\frac{e^{mh}}{1+e^{mh}},\;m=0,\pm 1,\pm 2,\ldots . \end{array}$$

Also, the* n*th derivative of the function *f* at some points *x*_*m*_ can be approximated [24]:

(27) δm,j(0)  =S(m,h)∘ψ(x)x=xj=1,m=j,0,m≠j,

(28) δm,j(1) =ddψS(m,h)∘ψ(x)x=xj =1h0,m=j,−1j−mj−m,m≠j,

(29) δm,j(2) =d2dψ2S(m,h)∘ψ(x)x=xj =1h2−π23,m=j,−2−1j−mj−m2,m≠j.

## 3. Convergence Rate Analysis 

### 3.1. Haar Functions

**Theorem 1 .**

Assume that *y*(*t*) ∈ *L* ^2^(*R*) with the bounded first derivative on (0,1); then, the error norm at *M*th level satisfies the following inequality:

(30) $$\begin{array}{c} \left\|e_{M}\left(t\right)\right\|\leq \sqrt{\frac{K}{7}}C2^{(-3/2)M}, \end{array}$$

where *K*, *C* are some real constants [28, 29].

**Proof**

The error at *M*th level may be defined as

(31) eMt =yt−yMt=∑i=2M+1+1∞cihi(t),eM(t)2=∫−∞∞∑i=2M+1+1∞cihi(t),∑q=2M+1+1∞cqhq(t)=∑i=2M+1+1∞ ∑q=2M+1+1∞cicq∫−∞∞hi(t)hq(t)dt,eM(t)2 ≤∑i=2M+1+1∞ci2,

where *y*_*M*_(*t*) = ∑_*i*=1_^2^*M*+1^^ *c*_*i*_ *h*_*i*_(*t*). But |*c*_*i*_ | ≤*C*2^−3*i*/2^max⁡|*y*′(*l*)|, where *C* = ∫_0_^1^ | *th*_2_(*t*) | *dt* and *l* ∈ (*k*2^−*m*^, (*k* + 1)2^−*m*^). Then

(32) eM(t)2≤∑i=2M+1+1∞KC22−3i,eM(t)2≤KC2172−3M,eM(t)≤K7C2−(3/2)M,

where *y*′(*t*) ≤ *K*  ∀*t* ∈ (0,1) and *K* is positive constant.

### 3.2. Sinc Functions

The following theorem for which the proof can be found in [21] shows that the convergence rate of Sinc approximation is exponential.

**Definition 2 .**

Let *H* ^2^(*D*_*E*_) be the class of functions *f* which are analytic in *D*_*E*_ (the eye-shaped domain defined in (21)) satisfy

(33) $$\begin{array}{c} \int_{\psi^{-1}(p+T)}\left|f\left(z\right)\right|dz⟶0,\;x⟶\pm \infty , \end{array}$$

where *T* = {*iq* : |*q* | <*d* ≤ *π*/2} and the function *f* satisfies the following equation on the boundary of *D*_*E*_:

(34) $$\begin{array}{c} N\left(f\right)=\int_{\partial D}\left|f\left(z\right)dz\right|<\infty . \end{array}$$

**Theorem 3 .**

Assume that *fψ*′ ∈ *H* ^2^(*D*_*E*_); then, for all *z* ∈ (0,1),

(35) Ef,hz=f(z)−∑k=−∞∞f(kh)S(k,h)∘ψ(z)≤N(fψ′)2πdsinh⁡(πd/h)≤2N(fψ′)πde−πd/h.

Moreover, if |*f*(*z*) | ≤*Ce* ^−*α*|*ψ*(*z*)|^,  *z* ∈ Γ for some positive constants *C*, *α* if the selection $h=\sqrt{\pi d/\alpha N}\leq 2\pi d/\ln(2)$, then

(36) $$\begin{array}{c} \left|f\left(z\right)-\overset{N}{\underset{k=-N}{\sum }}f\left(kh\right)S\left(k,h\right)\circ \psi \left(z\right)\right|\leq K\sqrt{N}e^{\left(-\sqrt{\pi d\alpha N}\right)}, \end{array}$$

where *K* depends only on *f*, *d*, and *α*.

## 4. Haar-Sinc Collocation Method

A discrete approximation to the (∂^2^ *y*/∂*t* ^2^)(*x*, *t*) can be expanded into 2*n* + 1 Sinc functions and *k* Haar functions as

(37) $$\begin{array}{c} \frac{\partial^{2}y_{n,k}}{\partial t^{2}}\left(x,t\right)=\overset{n}{\underset{i=-n}{\sum }}\;\overset{k-1}{\underset{j=0}{\sum }}c_{ij}S_{i}\left(x\right)h_{j}\left(t\right). \end{array}$$

**Lemma 4 .**

Let *x*_*m*_ be Sinc collocation points, given in (26). Then the following relations hold:

(38) ∂yn,k∂t(xm,t)=g1(xm)+∑j=0k−1 ∑l=0k−1cmlJljhj(t),yn,kxm,t=g0(xm)+g1(xm)t +∑j=0k−1 ∑l=0k−1cmlJlj2hj(t),∂2yn,k∂x2xm,t=∂2g0(x)+g1(x)t∂x2xm +∑i=−nn ∑j=0k−1 ∑l=0n−1wim(2)cilJlj2hj(t),

where

(39) $$\begin{array}{c} J_{lj}=J_{k\times k}\left[l,j\right],\\ w_{im}^{\left(2\right)}=\psi^{\prime \prime }\left(x_{m}\right)\delta_{i,m}^{\left(1\right)}+{\left(\psi^{\prime }\left(x_{m}\right)\right)}^{2}\delta_{i,m}^{\left(2\right)}. \end{array}$$

**Proof**

Employing (2), (14), (27), and (37) we have

(40) ∂yn,k∂txm,t=g1(xm)+∫0t∑i=−nn ∑j=0k−1cijSi(xm)hj(t)dt=g1(xm)+∫0t∑i=−nn ∑j=0k−1ci,jδi,m(0)hj(t)dt=g1(xm)+∫0t∑j=0k−1cmjhj(t)dt=g1(xm)+∑j=0k−1 ∑l=0k−1cmlJljhj(t).

Also, using (40) we get

(41) yn,kxm,t=g0(xm)+∫0t∂y∂t(x,t)dtxm=g0(xm)+∫0tg1(xm)dt +∫0t∑j=0k−1 ∑l=0k−1cmlJljhj(t)dt=g0(xm)+g1xmt+∑j=0k−1 ∑l=0k−1cmlJlj2hj(t).

In addition, using (28), (29), and (41) we have

(42) ∂2yn,k∂x2xm,t = ∂2g0(x)+g1(x)t∂x2xm︷A  +∑i=−nn ∑j=0k−1 ∑l=0k−1cilJlj2d2Sixdx2x=xmhj(t) =A+∑i=−nn ∑j=0k−1 ∑l=0k−1cilJlj2ψ′′xddϕSix+ψ′x2ddϕ2Si(x)x=xmhj(t) =A+∑i=−nn ∑j=0k−1 ∑l=0k−1cilJlj2ψ′′xmδi,m(1)+ψ′xm2δi,k(2)hj(t),

which completes our proof.

The residual *R*_*n*,*k*_(*x*, *t*) for (4) can be written as

(43) Rn,kx,t=∂2yn,k∂t2x,t+ρ+σ∂yn,k∂t(x,t) +ρσyn,k(x,t)−η2∂2yn,k∂x2(x,t)−f(x,t).

The equations for obtaining the (2*n* + 1)*k* coefficients {*c*_*ij*_}_*i*=−*n*⋯*n*_^*j*=0⋯*k*−1^ arise from equalizing *R*_*n*,*k*_(*x*, *t*) to zero at 2*n* + 1 Sinc points and *k* Haar points are defined by

(44) $$\begin{array}{c} x_{m}=\psi^{-1}\left(mh\right)=\frac{e^{mh}}{1+e^{mh}},\;m=0,\pm 1,\pm 2,\ldots ,\pm n,\\ t_{i}=\frac{2i-1}{2k},\;i=1,2,\ldots ,k. \end{array}$$

By substitution collocation points in *R*_*n*,*k*_(*x*, *t*) and equalizing to zero we have

(45) $$\begin{array}{c} R_{n,k}\left(x_{m},t_{i}\right)=0,\;m=-n,\ldots ,n,\;i=1,2,\ldots ,k. \end{array}$$

Equation (45) gives (2*n* + 1)*k* linear algebraic equations which can be solved for the unknown coefficients {*c*_*ij*_}_*i*=−*n*⋯*n*_^*j*=0⋯*k*^ by using the Newton's method. Consequently, *y*(*x*, *t*) given in (4) can be calculated.

## 5. Illustrative Examples

In this section, we apply the proposed method for solving (4) and show the efficiency of the method with the numerical results of some examples. In all examples we choose $h=\pi /\sqrt{2n}$.

**Example 1 .**

Consider the linear telegraph equation [1]:

(46) $$\begin{array}{c} \frac{\partial^{2}y}{\partial x^{2}}-4e^{-2t}\sinh\left(x\right)=\frac{\partial^{2}y}{\partial t^{2}}+4\frac{\partial y}{\partial t}+y, \end{array}$$

with the following initial and boundary conditions:

(47) $$\begin{array}{c} y\left(x,0\right)=\sinh\left(x\right),\;\;y_{t}\left(x,0\right)=-2\sinh\left(x\right),\\ y\left(0,t\right)=0,\;\;y\left(1,t\right)=e^{-2t}\sinh\left(1\right). \end{array}$$

The exact solution to this problem is

(48) $$\begin{array}{c} y\left(x,t\right)=e^{-2t}\sinh\left(x\right). \end{array}$$

Table 1 shows the absolute error function |*y*_exact_(*x*, *t*) − *y*_*n*,*k*_(*x*, *t*)| obtained by the present method with *n* = 3 and different values of *k*.

**Figure 1:**
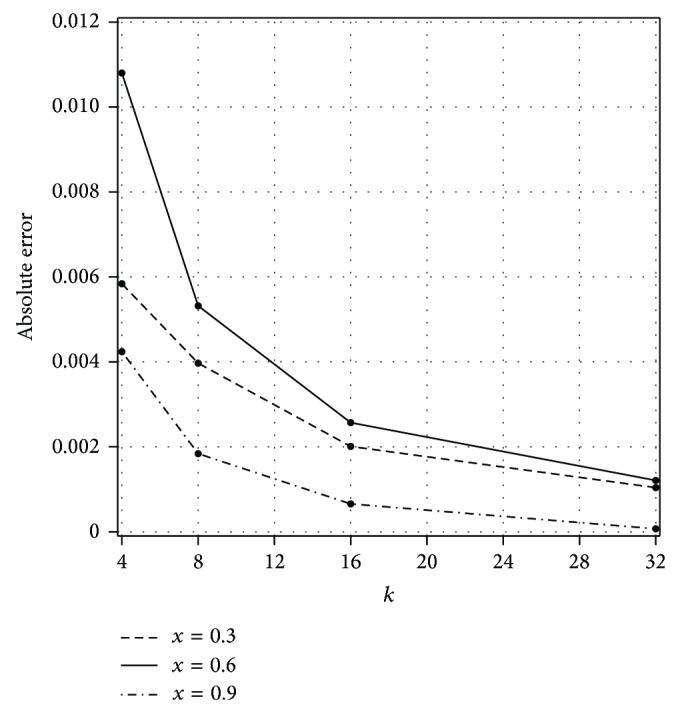
Plot of the absolute errors for different values of *k* with *t* = 0.5 and *n* = 3 for Example 1.

Also, Figure 1 displays the convergence rate of our method with *k* = 4, 8, 16, 32 for *t* = 0.5. It is seen from the Figure 1 that for each fixed point (*x*, *t*) the absolute errors get smaller and smaller as *k* increases. Furthermore, we can see that the presented method provides accurate results even by using *n* = 3.

**Figure 2:**
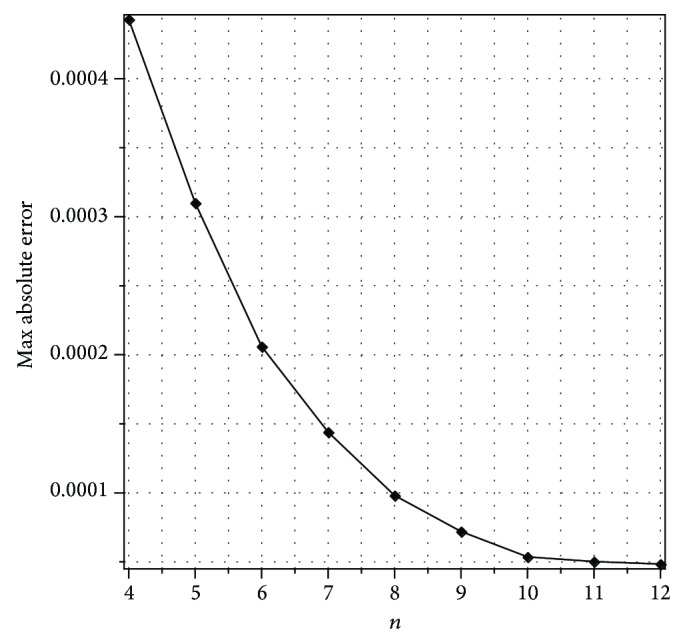
Plot of the maximum absolute errors for different values of *n* with *t* = 0.5 and *k* = 8 for Example 1.

The maximum absolute errors for *k* = 8 and different values of *n* (Sinc collocation points) are shown graphically in Figure 2 for *t* = 0.5. In Figure 2, we observe that the values of maximum absolute error decay exponentially as expected from Theorem 3.

**Example 2 .**

Consider the linear telegraph equation [5]:

(49) $$\begin{array}{c} \frac{\partial^{2}y}{\partial x^{2}}=\frac{\partial^{2}y}{\partial t^{2}}+4\frac{\partial y}{\partial t}+4y, \end{array}$$

with the following initial and boundary conditions:

(50) $$\begin{array}{c} y\left(x,0\right)=e^{x},\;\;y_{t}\left(x,0\right)=-e^{x},\\ y\left(0,t\right)=e^{-t},\;\;y\left(1,t\right)=e^{1-t}. \end{array}$$

The exact solution is given by

(51) $$\begin{array}{c} y\left(x,t\right)=e^{x-t}. \end{array}$$

Table 2 shows the absolute error values using the proposed method with *n* = 3 and *k* = 8,16,32.

**Figure 3:**
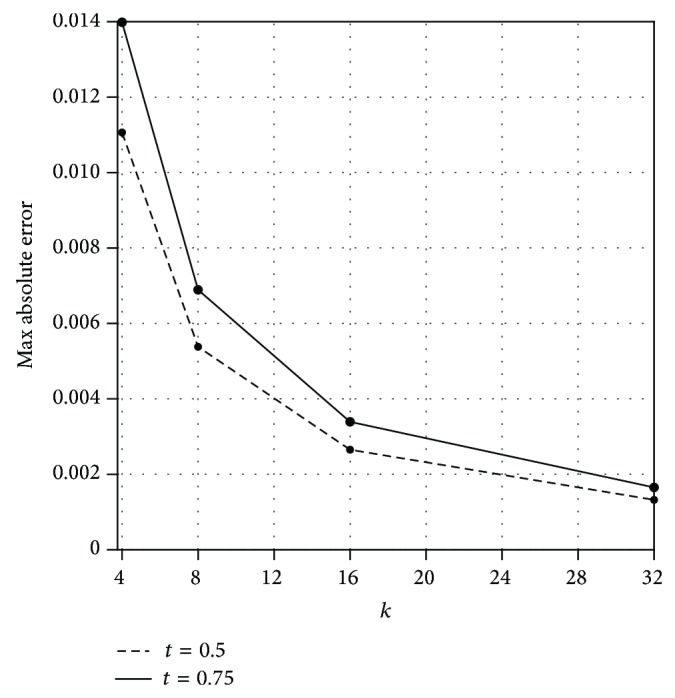
Plot of the maximum absolute errors for different values of *k* with *t* = 0.5,0.75 and *n* = 3 for Example 2.

Figure 3 displays the values of maximum absolute error with *k* = 4,8, 16,32 for *t* = 0.5,0.75. This figure demonstrates the validity and applicability of the present technique for this problem. Also, we observe that the convergence rate of our method for *t* = 0.75 is lower than *t* = 0.5.

**Figure 4:**
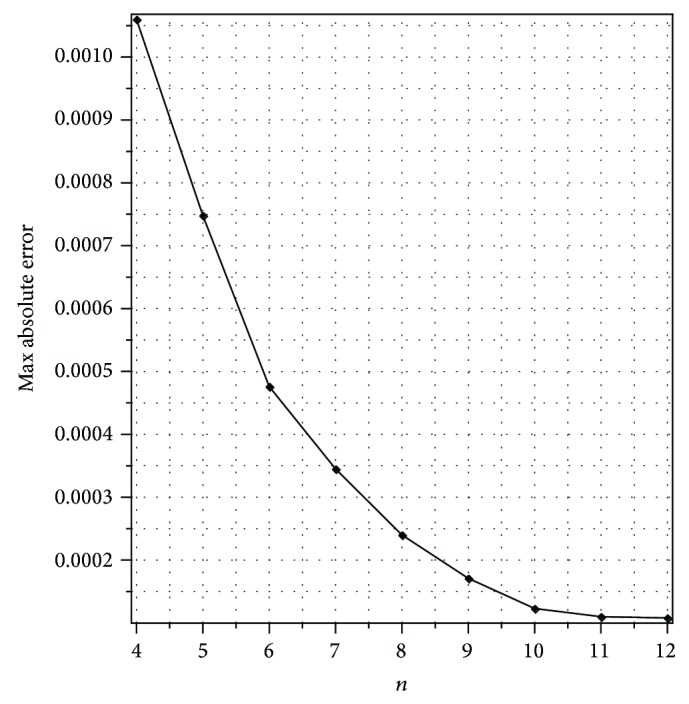
Plot of the maximum absolute errors for different values of *n* with *t* = 0.75 and *k* = 8 for Example 2.

Figure 4 displays the convergence rate of our method for different values of *n* for *t* = 0.75 and *k* = 8. We see from Figure 4 that our method is in good agreement with the actual rate of convergence and the values of maximum absolute error decay exponentially.

**Example 3 .**

Consider the nonlinear telegraph equation [5]:

(52) $$\begin{array}{c} \frac{\partial^{2}y}{\partial x^{2}}=\frac{\partial^{2}y}{\partial t^{2}}+2\frac{\partial y}{\partial t}+y^{2}-e^{2x-4t}+e^{x-2t}, \end{array}$$

with the following initial and boundary conditions:

(53) $$\begin{array}{c} y\left(x,0\right)=e^{x},\;\;y_{t}\left(x,0\right)=-2e^{x},\\ y\left(0,t\right)=e^{-2t},\;\;y\left(1,t\right)=e^{1-2t}. \end{array}$$

The exact solution is given by

(54) $$\begin{array}{c} y\left(x,t\right)=e^{x-2t}. \end{array}$$

**Figure 5:**
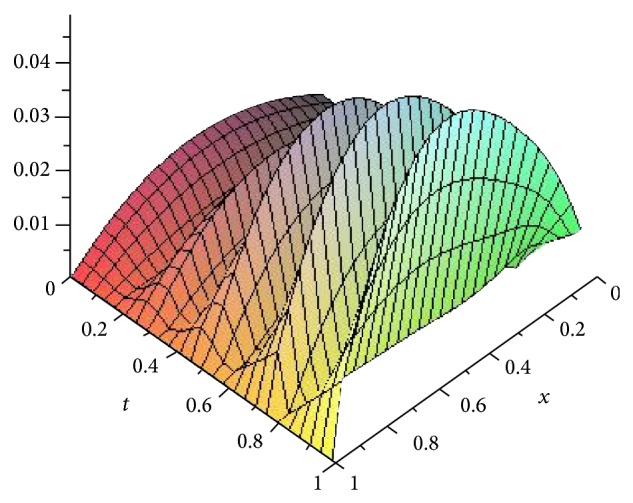
Plot of the absolute error *e*(*x*, *t*) = |*y*(*x*, *t*) − *y*_*n*,*k*_(*x*, *t*)| with *n* = 3,  *k* = 4 for Example 3.

**Figure 6:**
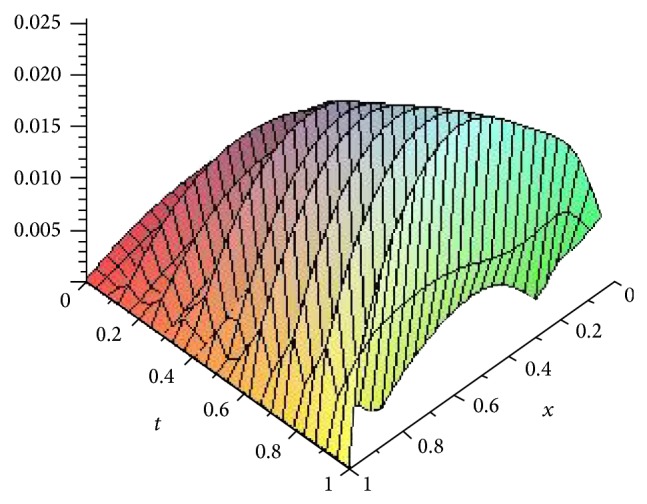
Plot of the absolute error *e*(*x*, *t*) = |*y*(*x*, *t*) − *y*_*n*,*k*_(*x*, *t*)| with *n* = 3,  *k* = 8 for Example 3.

**Figure 7:**
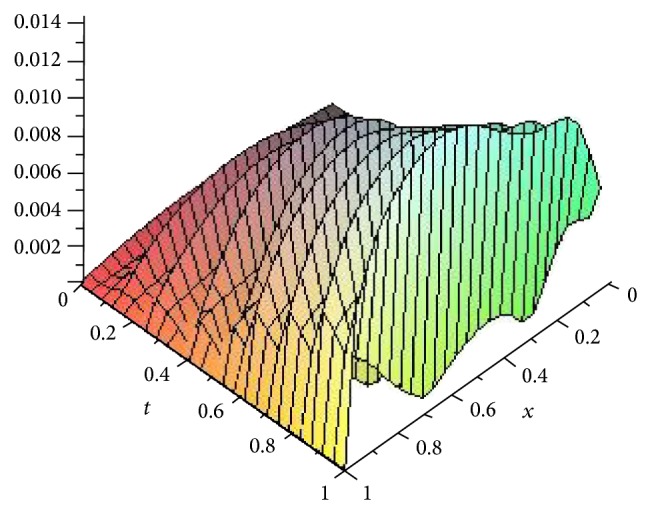
Plot of the absolute error *e*(*x*, *t*) = |*y*(*x*, *t*) − *y*_*n*,*k*_(*x*, *t*)| with *n* = 3,  *k* = 16 for Example 3.

**Figure 8:**
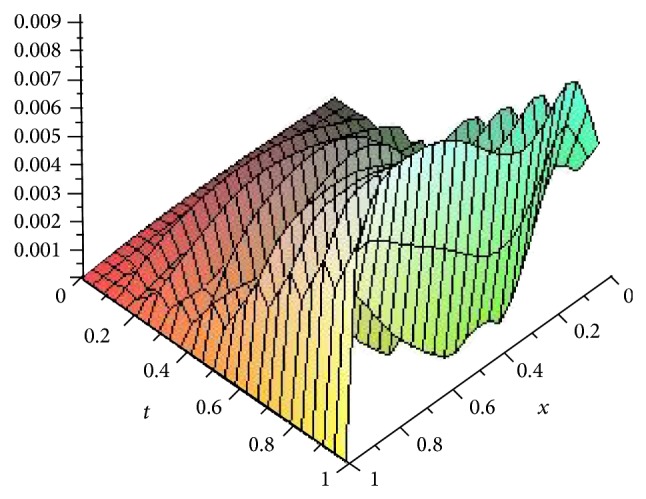
Plot of the absolute error *e*(*x*, *t*) = |*y*(*x*, *t*) − *y*_*n*,*k*_(*x*, *t*)| with *n* = 3,  *k* = 32 for Example 3.

Figures 5, 6, 7, and 8 show the absolute error function |*y*_exact_(*x*, *t*) − *y*_*n*,*k*_(*x*, *t*)| obtained by the present method with *n* = 3 and *k* = 4,8, 16 and 32. We can see clearly that better accuracy can be achieved by increasing the number of Haar collocation points and using the arbitrary precision ability of Mathematica software, we are able to establish more accurate results.

**Example 4 .**

Consider the numerical computation of the nonlinear telegraph equations [31]:

(55) $$\begin{array}{c} \frac{\partial^{2}y}{\partial x^{2}}=\frac{\partial^{2}y}{\partial t^{2}}+2\frac{\partial y}{\partial t}-y^{3}+y. \end{array}$$

The exact solution is given by

(56) $$\begin{array}{c} y\left(x,t\right)=\frac{1}{2}+\frac{1}{2}\tanh\left(\frac{x}{8}+\frac{3t}{8}+5\right). \end{array}$$

We extract the initial and boundary conditions from the exact solution.  Table 3 shows the comparison of the *L*_*∞*_ error between approximations obtained by the radial basis functions (IMQ, TSP) [30] and the Haar-Sinc collocation method with *n* = 3 and *k* = 8,16,32. The results obtained in the table show that the *L*_*∞*_ error between the numerical and the exact solution can be reduced by increasing the values of *k*.

## 6. Conclusion

A numerical method for solving the telegraph equations based on the combination of two orthogonal Haar and Sinc functions was proposed. Also, [32, 33] have previously applied the combination of the Sinc functions with the other different groups of orthogonal Legendre and Chebyshev polynomials for solving similar PDEs. It is worth mentioning that since the convergence rate of the Sinc approximation is exponential, we expand the problem in space with the Sinc basis functions. Furthermore, according to the initial conditions of the problem and the Haar operational matrix for integration, the approximation in time was expanded via the elements of Haar functions. This method can be applied to solve similar problems in physics and provides a powerful alternative for physicians to investigate such types of nonlinear PDEs. The effectiveness of the method was examined via comparing the obtained results with the exact solutions. Based on the numerical results, it is obvious that it can more entirely simulate the global property of the exact solution and can provide more information about the structures of the problem. Also, the absolute error may be decreased if we take more collocation points.

## Figures and Tables

**Table 1:** The values of absolute error with *n* = 3 for Example 1.

*x*	*t*	|*y*_exact_(*x*, *t*) − *y*_*n*,*k*_(*x*, *t*)|		
		*k* = 8	*k* = 16	*k* = 32
0.3	0.25	2.72*e* − 3	1.31*e* − 3	6.56*e* − 4
	0.50	3.97*e* − 3	2.01*e* − 3	1.04*e* − 3
	0.75	4.65*e* − 3	2.34*e* − 3	1.19*e* − 3

0.6	0.25	3.72*e* − 3	1.75*e* − 3	8.41*e* − 4
	0.50	5.32*e* − 3	2.57*e* − 3	1.21*e* − 3
	0.75	6.08*e* − 3	2.81*e* − 3	1.18*e* − 3

0.9	0.25	1.51*e* − 3	6.84*e* − 4	2.89*e* − 4
	0.50	1.84*e* − 3	6.57*e* − 4	7.14*e* − 5
	0.75	1.61*e* − 3	1.81*e* − 4	5.27*e* − 5

**Table 2:** The values of absolute error with *n* = 3 and *k* = 8,16,32 for Example 2.

*x*	*t* = 0.5			*t* = 0.75		
	*k* = 8	*k* = 16	*k* = 32	*k* = 8	*k* = 16	*k* = 32
0.1	1.22*e* − 3	4.16*e* − 4	2.12*e* − 5	1.17*e* − 3	3.83*e* − 4	1.32*e* − 4
0.2	2.85*e* − 3	1.30*e* − 3	5.44*e* − 4	3.38*e* − 3	1.38*e* − 3	3.98*e* − 4
0.3	4.22*e* − 3	2.08*e* − 3	1.03*e* − 3	5.35*e* − 3	2.60*e* − 3	1.23*e* − 3
0.4	5.06*e* − 3	2.52*e* − 3	1.28*e* − 3	6.50*e* − 3	3.24*e* − 3	1.62*e* − 3
0.5	5.38*e* − 3	2.65*e* − 3	1.32*e* − 3	6.89*e* − 3	3.39*e* − 3	1.65*e* − 3
0.6	5.25*e* − 3	2.55*e* − 3	1.24*e* − 3	6.64*e* − 3	3.17*e* − 3	1.46*e* − 3
0.7	4.64*e* − 3	2.22*e* − 3	1.04*e* − 4	5.77*e* − 3	2.64*e* − 3	1.09*e* − 3
0.8	3.47*e* − 3	1.59*e* − 3	6.72*e* − 4	4.13*e* − 3	1.69*e* − 3	4.83*e* − 4
0.9	1.66*e* − 3	6.06*e* − 4	8.98*e* − 5	1.68*e* − 3	3.76*e* − 4	3.02*e* − 4

**Table 3:** Results for the value of *L*_∞_ error obtained by Haar-Sinc collocation method with *n* = 3 and *k* = 8,16,32 for Example 4.

*t*	*L*_∞_− error [30]		*L*_∞_− error (present method)		
	TPS	IMQ	*k* = 8	*k* = 16	*k* = 32
0.5	1.014*e* − 5	3.599*e* − 6	1.725*e* − 6	6.598*e* − 7	7.91*e* − 8
1.0	1.668*e* − 5	6.375*e* − 7	4.237*e* − 6	3.440*e* − 6	3.93*e* − 7
1.5	1.087*e* − 5	1.156*e* − 6	7.089*e* − 6	6.578*e* − 6	6.31*e* − 7
2.0	3.633*e* − 6	6.750*e* − 7	7.248*e* − 6	6.890*e* − 6	7.13*e* − 7
3.0	2.159*e* − 6	4.154*e* − 7	7.873*e* − 6	7.869*e* − 6	7.18*e* − 7
